# Supplementary material for: Association between oral microbiome and seven types of cancers in East Asian population: a two-sample Mendelian randomization analysis
Source: Front Mol Biosci. 2023 Nov 21;10:1327893. doi: 10.3389/fmolb.2023.1327893 (PMC10702768; doi:10.3389/fmolb.2023.1327893)
Supplement: Supplementary file 1 [file DataSheet1.ZIP › Supplementary Materials/MR plots of saliva/saliva═╝/colorectal cancer/pheno.3020_to_colorectal cancer_leave_one_out.pdf]

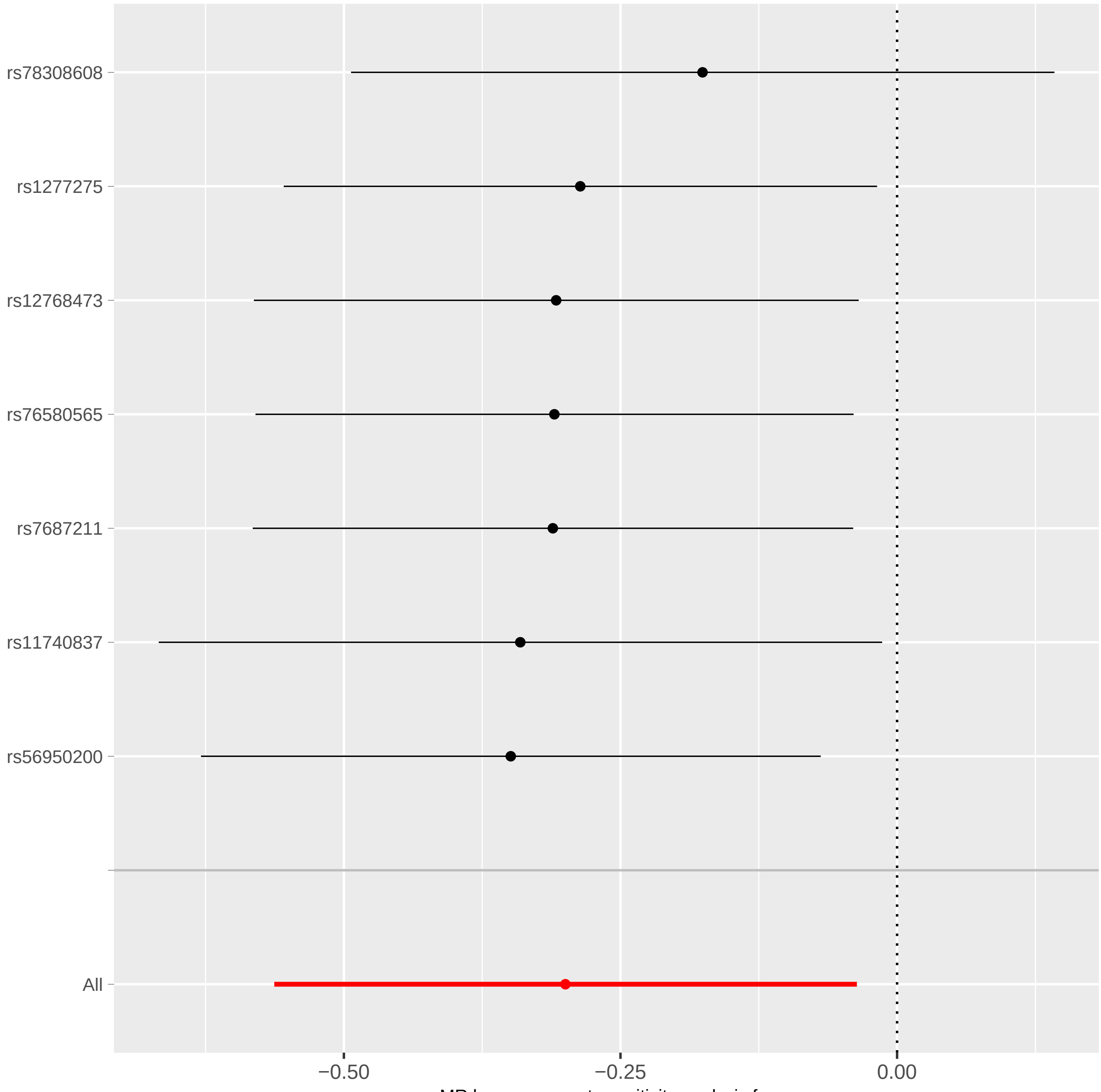

MR leave−one−out sensitivity analysis for
'pheno.3020.assoc.linear.gz.raw.gz' on 'Colorectal cancer || id:bbj−a−107'
